# Prevalence of Fungemia in People with HIV: A Systematic Review and Meta-Analysis

**DOI:** 10.3390/microorganisms14010225

**Published:** 2026-01-19

**Authors:** Asta Maria Blom Nielsen, Kristiana Alexandrova Nikolova, Tea Nynne Sanders, Ask Bock, Moises Alberto Suarez-Zdunek, Susanne Dam Nielsen

**Affiliations:** 1 Viroimmunology Research Unit, Department of Infectious Diseases, Copenhagen University Hospital—Rigshospitalet, 2100 Copenhagen, Denmark; 2 Department of Clinical Medicine, Faculty of Health and Medical Sciences, University of Copenhagen, 2200 Copenhagen, Denmark

**Keywords:** viruses, infections, HIV, fungemia, fungi, prevalence, meta-analysis, systematic review

## Abstract

Prior to the introduction of antiretroviral therapy (ART), people with HIV (PWH) had high risk of fungemia. No systematic review has assessed the prevalence of fungemia in PWH after the introduction of combination ART in 1996. The primary objective of this systematic review was to determine the prevalence of fungemia in adult PWH after 1996. Furthermore, we aimed to compare the prevalence of fungemia in different ART time periods to determine geographic differences and fungal pathogen distribution. A systematic literature search was performed on 7 March 2025 across six databases and the study quality was assessed using the Newcastle–Ottawa scale. Prevalence estimates were extracted, and a meta-analysis was performed using a random effects model. Twelve studies comprising 27,729 PWH were included. The overall pooled prevalence in PWH was 3.3% (95% CI: 1.53; 4.96%, I^2^ = 98.9%). The most common pathogen to cause fungemia was *Talaromyces marneffei* with a prevalence of 4.8%, although this pathogen was limited to studies from Asia. The highest prevalence of fungemia in PWH was 6.8% in Asia. The prevalence of fungemia was 5.8% between July 1996–September 2015 and 1.0% between September 2015–January 2025, but the difference was not statistically significant (*p* = 0.273). However, all findings were limited by very low certainty of evidence and should be interpreted with caution. In conclusion, our findings suggest that fungemia persists among PWH despite ART, especially in Asia. Given the limited available evidence, it was not possible to determine whether the prevalence of fungemia changed following the change in ART treatment guidelines in September 2015. The protocol is registered in PROSPERO (CRD420251005081).

## 1. Introduction

Fungemia is defined as the presence of fungal pathogens in the bloodstream and is one of the most severe manifestations of fungal infections [1]. A wide spectrum of fungal pathogens can cause fungemia, and the clinical manifestations of fungemia are often nonspecific, leading to delayed diagnosis and treatment [2]. Common for all types of fungemia are high mortality rates and severe illness [3,4].

Fungal infections are opportunistic and can lead to serious illness during immunodeficiency. Before the introduction of combination antiretroviral therapy (ART) in 1996, people with HIV (PWH) were particularly susceptible to fungal infections. Due to new treatment guidelines in 2015 recommending that all PWH begin ART regardless of immune status and increased focus on early detection, severe immunodeficiency among PWH has become rare [5,6,7]. As a result, the burden of fungemia in PWH has decreased in many parts of the world. However substantial challenges remain in the global management and treatment of HIV, such as limited availability of specialized HIV care and inadequate healthcare structures [8,9]. As a consequence of this, invasive fungal infections continue to affect PWH, especially in regions where access to treatment and diagnostic tools are lacking [9].

In 2022, the World Health Organization (WHO) published the fungal priority pathogen list to systematically address the growing global health threat posed by fungal infections [10]. This was partly due to increased antifungal resistance observed in certain fungal species leading to a serious health threat in all individuals with invasive fungal infections [11,12]. Thus, gaining new insights into the burden of fungemia in PWH is crucial to understand the challenges posed by fungal pathogens.

Although fungemia is a severe and life-threatening condition, there are no systematic reviews of studies reporting the prevalence of fungemia in PWH since the introduction of ART in 1996. The primary objective of this systematic review and meta-analysis was to describe the overall prevalence of fungemia in PWH after 1996. The secondary objectives were to compare the prevalence of fungemia in PWH in different ART time periods (July 1996–September 2015 and September 2015–January 2025), to determine geographic differences, and to determine fungal pathogen distribution in PWH with fungemia.

## 2. Materials and Methods

This systematic review and meta-analysis was performed according to Joanna Briggs Institute (JBI) guidelines for prevalence and incidence studies and reported according to the Preferred Reporting Items for Systematic Review and Meta-Analysis (PRISMA) guidelines [13,14]. The protocol was preregistered on 5 March 2025 in the Prospective Register for Systematic Reviews (PROSPERO) (CRD420251005081).

### 2.1. Data Sources and Searches

A systematic literature search across the PubMed MEDLINE, EMBASE, CINAHL, Web of Science, Cochrane Central Register of Controlled Trials, and Scopus databases was conducted on 7 March 2025. Based on the study objectives, the following two key terms were identified: “people with HIV” and “fungemia”. These key terms formed the foundation for the search terms. For PubMed MEDLINE, the search included a MeSH term search and free-text word search. The MeSH term search in PubMed MEDLINE included the following terms: (“HIV”[Mesh] OR “HIV Infections”[Mesh]) AND (“Invasive Fungal Infections”[Mesh]). The free-text word search in PubMed MEDLINE included the following terms: (“HIV seropositiv*” OR “HIV/AIDS” OR “HIV infection*” OR “human immunodeficiency virus” OR “HIV” OR “PLWH” OR “PWH” OR “AIDS” OR “Acquired immuno deficiency syndrome” OR “Acquired immune-deficiency syndrome”) AND (“fungal septi*” OR “fungal bloodstream infection*” OR “fungemia” OR “fungaemia” OR “invasive fungal infection*” OR “invasive myco*” OR “systemic myco*” OR “Disseminated fungal infection*” OR “invasive mold infection*” OR “Candidemia” OR (“Histoplasmosis” OR “Cryptococcus” OR “Coccidiomycosis” OR “Aspergillus” AND “blood”)). Search strings for all databases are available in the Supplementary Materials (Table S1).

### 2.2. Inclusion and Exclusion Criteria

Studies were included if they fulfilled the following inclusion criteria: (1) original peer-reviewed studies of observational design (cohort, case–control, and cross-sectional studies) or randomized controlled trials describing the prevalence of fungemia in PWH; (2) studies written in English; (3) adult PWH ≥ 18 years of age; (4) fungus isolated from peripheral or central blood and culture-proven detection of the fungus. Studies were excluded if they (1) were case reports, case series, any type of reviews, comments, editorials, experimental studies, grey literature, letters, or communications, unless they reported on errors or withdrawn publications considered in our review; (2) did not report the prevalence of fungemia; (3) collected data before 1996; or (4) reported fungemia detected by methods other than blood culture, including antigen detection and PCR.

### 2.3. Study Selection

**Figure 1:**
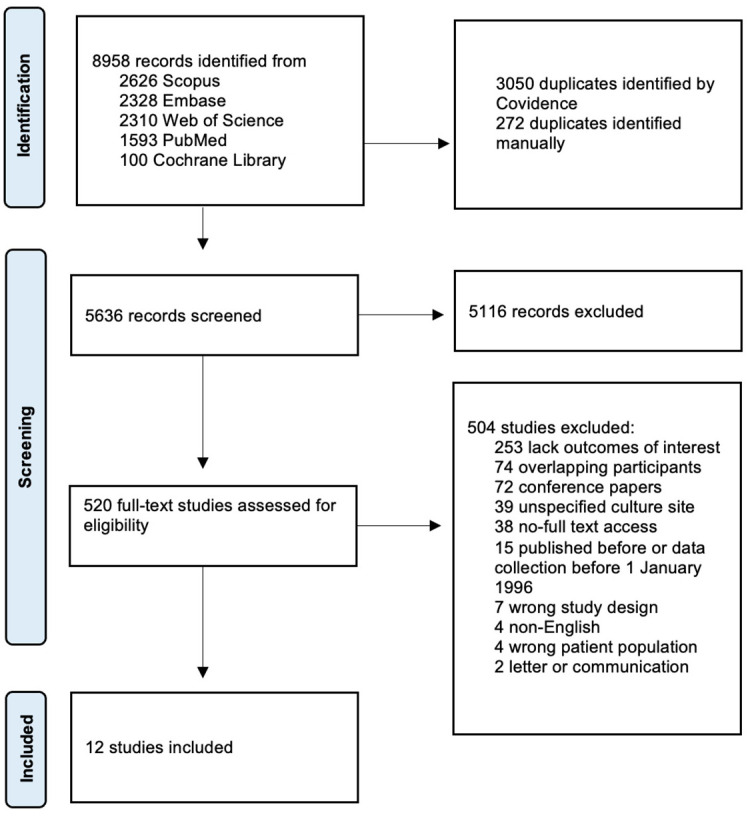
PRISMA 2020 flow diagram of the search and selection process.

Identified studies were transferred to Covidence systematic review software (Veritas Health Innovation, Melbourne, Australia) [15]. All studies were independently screened by two investigators (AN, KN, or TS) based on the established inclusion and exclusion criteria. Any disagreements between individual judgements were resolved by involving a third investigator. If data were missing or unclear, corresponding authors were contacted by e-mail for clarification. The study selection and screening process is summarized in the PRISMA flow diagram (Figure 1).

### 2.4. Data Extraction

Data extraction was performed independently by two investigators (AN and KN) using Covidence systematic review software [15]. Extracted data included authors, study period, study design, geography, study sample size, cases of fungemia, and pathogen distribution. We extracted all available results for each outcome domain.

### 2.5. Quality Assessment

Quality assessment of the included studies was performed using the Newcastle–Ottawa scale (NOS) [16] by two investigators (AN and TS) independently based on selection of study groups, the comparability of the groups, and the outcome of interest. However, because our primary aim was prevalence of fungemia, assessment of the quality of comparability and statistical tests of the outcome were not applicable as this review focused on a binary outcome. Yielding a maximum of 7 stars possible in our ratings, the study quality was classified as low (<5), moderate (5), or high (>5). Furthermore, the JBI checklist for prevalence studies was used as supplementary tool to evaluate domains not covered in NOS [17].

### 2.6. Certainty of Evidence

Grades of recommendation, assessment, development, and evaluation (GRADE) were used to assess the quality of the body of evidence [18] by one investigator (AN). Here, we assessed the certainty of evidence by evaluating five key domains: study design limitations, inconsistency of results, indirectness, imprecision, and publication bias.

### 2.7. Data Synthesis and Analysis

All prevalences in this study are period prevalences calculated as the proportion of PWH with fungemia after HIV diagnosis. The pooled prevalence of fungemia in PWH was calculated using a random effects model assuming that each study estimates a different effect, with *p* ≤ 0.05 being considered statistically significant. The results were presented using a forest plot, including the corresponding 95% confidence intervals (CI). We used I^2^ statistics to quantify heterogeneity, and I^2^ > 50% was considered to indicate substantial heterogeneity. Publication bias was evaluated using funnel plots and Egger’s test. The source of heterogeneity was investigated with a Baujot plot, leave-one-out analysis, and influential study diagnostics. Sensitivity analyses were conducted according to quality assessment scores. In our subgroup analyses conducted with studies from different time periods, the division of time periods was determined based on the official change in WHO treatment guidelines on 30 September 2015 [19] and the introduction of combination ART in July 1996 [20]. Subgroup analyses were conducted for the following variables: geographic regions (continents); fungal pathogens and different ART time periods (July 1996–September 2015 and September 2015–January 2025). Some subgroup analyses presented in the protocol were not conducted due to insufficient data. All analyses were performed using the software R (version 4.4.3).

## 3. Results

### 3.1. Study Selection and Characteristics

The initial search retrieved 8958 studies, of which 3322 were duplicates and were removed. After title and abstract screening, 520 studies were eligible for full-text review, of which 12 studies met the inclusion criteria and were included in the systematic review [21,22,23,24,25,26,27,28,29,30,31,32]. All included studies were cohort studies. The study selection process is visualized in the PRISMA flow diagram (Figure 1).

The studies provided data from 1996 to 2019. The total sample size across all included studies was 27,729 PWH ranging from 78 to 7575 participants in the individual studies. Studies from six different countries were included in the analysis. Characteristics of the included studies are summarized in Table 1.

### 3.2. Quality Assessment

The median NOS score was 4.5 (range: 4–6) stars (Table 2). The quality assessment revealed possible issues regarding selection and description of participants but showed high quality of detection of exposure and outcome. The quality assessment using the JBI checklist for prevalence studies is described in the Supplementary Materials (Table S2).

### 3.3. Overall Prevalence

**Figure 2:**
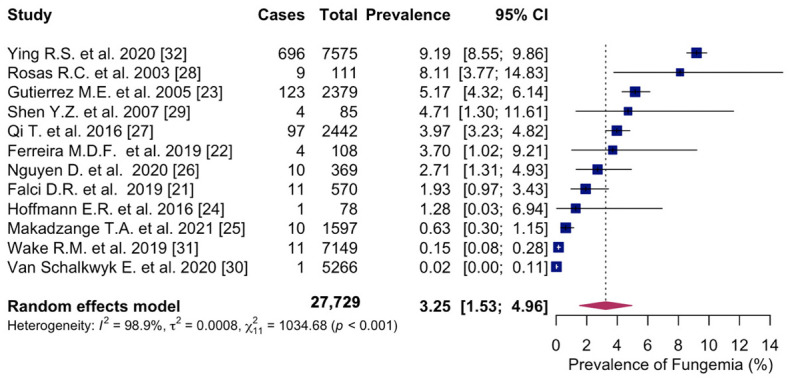
Forest plot of the pooled prevalence of fungemia in people with HIV, displaying the proportion of events per 100 observations. The proportions indicate the percentage of included people with HIV with a history of culture-proven fungemia in the time period. All estimates were conducted based on culture-proven cases of fungemia in people with HIV ≥ 18 years of age. CI: confidence interval. Blue square represents individual study prevalence. Black or white horizontal line represents confidence interval of each study. Dotted vertical line represents random effects estimate. Purple diamond shows the overall effects estimate. References [21,22,23,24,25,26,27,28,29,30,31,32].

The overall pooled prevalence of fungemia in PWH was 3.3% (95% CI: 1.53; 4.96%, 12 studies) with study-specific prevalence ranging from 0.02% to 9.2%. Considerable heterogeneity was observed across studies (I^2^ = 98.9%) (Figure 2).

### 3.4. Subgroup Analyses

#### 3.4.1. Geographic Regions

**Figure 3:**
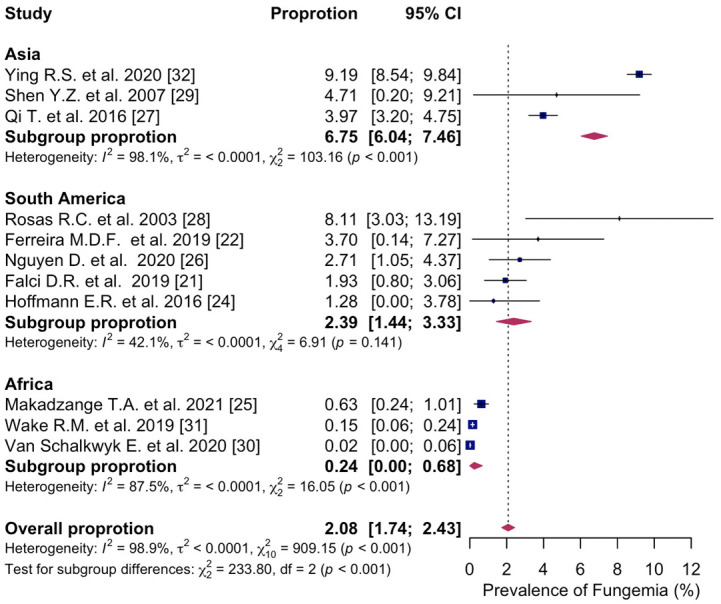
Forest plot of the pooled prevalence of fungemia in people with HIV in Asia; South America; Africa, displaying the proportion of events per 100 observations. The proportions indicate the percentage of included people with HIV with a history of culture-proven fungemia in the time period. All estimates were conducted based on culture-proven cases of fungemia in people with HIV ≥ 18 years of age. CI: confidence interval. Blue square represents individual study prevalence. Black or white horizontal line represents confidence interval of each study. Dotted vertical line represents random effects estimate. Purple diamond shows the overall effects estimate. References [21,22,24,25,26,27,28,29,30,31,32].

In subgroup analyses of the geographic region, sufficient data were available to estimate the prevalence of fungemia in Asia, South America, and Africa. Only one study reported data from North America, and no studies were identified from Europe. The highest prevalence of fungemia in PWH was in Asia, with a prevalence of 6.8% (95% CI: 6.04; 7.46%, I^2^ = 98.1%, three studies) [27,29,32], followed by 2.4% in South America (95% CI: 1.44; 3.33%, I^2^ = 42.1%, five studies) [21,22,24,26,28]. The prevalence of fungemia was lowest in Africa, at 0.2% (95% CI: 0.00; 0.68%, I^2^ = 88.7%, three studies) [25,30,31]. There was a statistically significant difference in the prevalence of fungemia across the geographic regions (*p* < 0.001) (Figure 3).

#### 3.4.2. Pathogen Distribution

**Figure 4:**
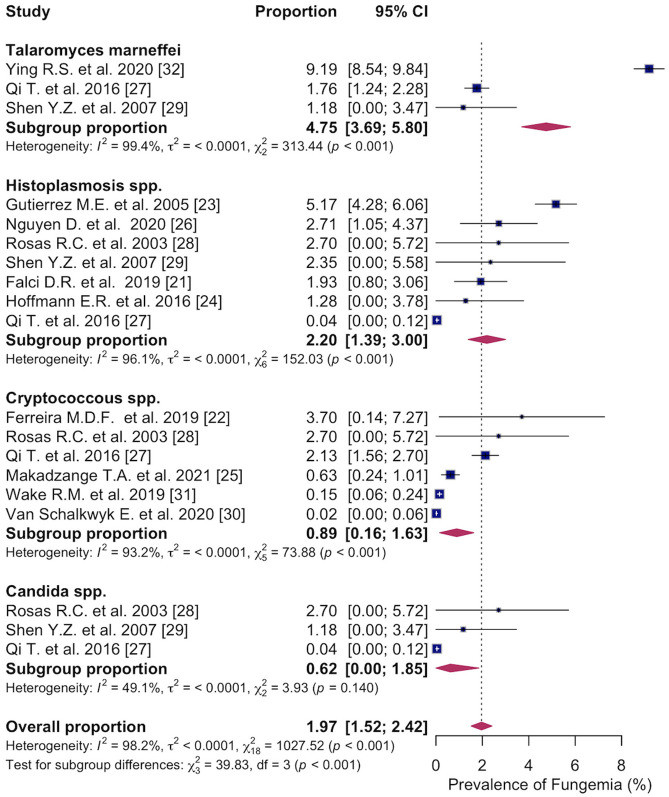
Forest plot of the pooled prevalence of fungemia in people with HIV caused by *Talaromyces marneffei*; *Histoplasma* spp.; *Cryptococcus* spp.; *Candida* spp., displaying the proportion of events per 100 observations. The proportions indicate the percentage of included people with HIV with a history of culture-proven fungemia in the time period. All estimates were conducted based on culture-proven cases of fungemia in people with HIV ≥ 18 years of age. CI: confidence interval. Blue square represents individual study prevalence. Black or white horizontal line represents confidence interval of each study. Dotted vertical line represents random effects estimate. Purple diamond shows the overall effects estimate. References [21,22,23,24,25,26,27,28,29,30,31,32].

Subgroup analyses of pathogen distribution showed that the most common fungal pathogen causing fungemia was *Talaromyces marneffei* with a prevalence of 4.8% (95% CI: 3.69; 5.80%, I^2^ = 99.4%, three studies) [27,29,32]. This was followed by *Histoplasma* spp. with a prevalence of 2.2% (95% CI: 1.39; 3.00%, I^2^ = 96.1%, seven studies) [21,23,24,26,27,28,29], *Cryptococcus* spp. with a prevalence of 0.9% (95% CI: 0.16; 1.63%, I^2^ = 93.2%, six studies) [22,25,27,28,30,31], and *Candida* spp. with a prevalence of 0.6% (95% CI: 0.00; 1.85%, I^2^ = 49.1%, three studies) [27,28,29]. The differences in distribution of the fungal pathogen causing fungemia were statistically significant (*p* < 0.001) (Figure 4).

#### 3.4.3. ART Time Periods

**Figure 5:**
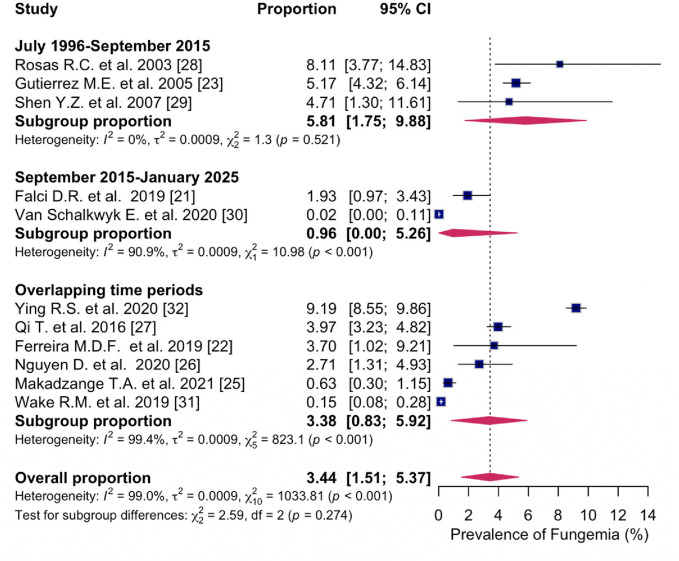
Forest plot of the pooled prevalence of fungemia in people with HIV between January 1996–September 2015; between September 2015–January 2025; overlapping time periods, displaying the proportion of events per 100 observations. The proportions indicate the percentage of included people with HIV with a history of culture-proven fungemia in the time period. All estimates were conducted based on culture-proven cases of fungemia in people with HIV ≥ 18 years of age. CI: confidence interval. Blue square represents individual study prevalence. Black or white horizontal line represents confidence interval of each study. Dotted vertical line represents random effects estimate. Purple diamond shows the overall effects estimate. References [21,22,23,25,26,27,28,29,30,31,32].

The pooled prevalence estimate of fungemia in PWH in studies published between July 1996–September 2015 was 5.8% (95% CI: 1.75; 9.88%, I^2^ = 0%, three studies) [23,28,29] and 1.0% (95% CI: 0.00; 5.26%, I^2^ = 90.9%, two studies) [21,30] in studies published between September 2015–January 2025. The prevalence of fungemia in studies overlapping the two time periods was 3.4% (95% CI: 0.83; 5.92%, I^2^ = 99.4%, six studies) [22,25,26,27,31,32]. The difference was not statistically significant (*p* = 0.273) (Figure 5).

### 3.5. Source of Heterogeneity

**Figure 6:**
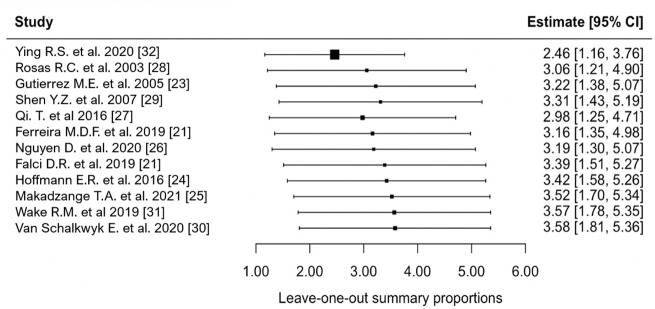
Leave-one-out forest plot of the included studies, displaying the proportion of events per 100 observations. The proportions indicate the percentage of included people with HIV with a history of culture-proven fungemia in the time period. All estimates were conducted based on culture-proven cases of fungemia in people with HIV ≥ 18 years of age. CI: confidence interval. Black square represents overall prevalence after the omitted study. Black horizontal line represents confidence interval of each study. References [21,23,24,25,26,27,28,29,30,31,32].

The Baujot plot revealed that Ying R.S. et al. 2020 [32] had substantial influence on the overall pooled prevalence and overall heterogeneity (Supplementary Materials; Figure S1). Furthermore, when investigating the individual z-scores of the included studies, the study had a z-score = 3.2, indicating that the study was considered an outlier. Our influential study diagnostics also showed that this study was deemed influential on the overall prevalence (Supplementary Materials; Figure S2). The leave-one-out method showed that Ying R.S. et al. 2020 [32] had remarkable influence on the overall pooled prevalence. When excluding the study, the estimate of the prevalence of fungemia was 2.5% (95% CI: 1.16; 3.76%, 11 studies) (Figure 6). None of the other studies had remarkable influence on the overall pooled estimate nor on the heterogeneity between studies.

**Figure 7:**
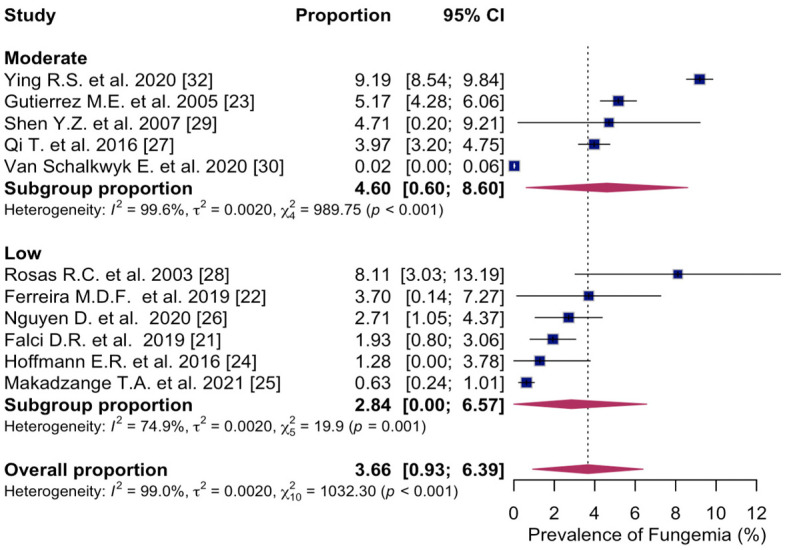
Forest plot of the pooled prevalence of fungemia in people with HIV in studies scoring moderate quality; low quality, displaying the proportion of events per 100 observations. The proportions indicate the percentage of included people with HIV with a history of culture-proven fungemia in the time period. All estimates were conducted based on culture-proven cases of fungemia in people with HIV ≥ 18 years of age. CI: confidence interval. Blue square represents individual study prevalence. Black or white horizontal line represents confidence interval of each study. Dotted vertical line represents random effects estimate. Purple diamond shows the overall effects estimate. References [21,22,23,24,25,26,27,28,29,30,32].

The subgroup analysis conducted based on the quality assessment score obtained via NOS showed that the prevalence of fungemia in PWH was highest in studies scoring moderate quality with a prevalence of 4.6% (95% CI: 0.60; 8.60%, I^2^ = 99.6%, five studies) [23,27,29,30,32] and 2.8% (95% CI: 0.00; 6.57%, I^2^ = 74.9%, six studies) [21,22,24,25,26,28] in studies scoring low quality (Figure 7).

### 3.6. Assesment of Publication Bias

The funnel plot of the included studies showed asymmetry (Supplementary Materials; Figure S3). Likewise, quantitative evaluation of the publication bias using Egger’s test revealed potential publication bias of the included studies (t = 2.64, *p* = 0.0246), suggesting that effect sizes are asymmetrically distributed.

### 3.7. Certainty of Evidence

All analyses in this systematic review and meta-analysis were rated as having very low certainty of evidence. Due to inclusion of only observational studies, the initial rating was low. Further downgrading to very low certainty of evidence was due to the presence of multiple studies with low quality assessments, as well as due to the indirectness, inconsistency, and imprecision of estimates across studies.

## 4. Discussion

This systematic review and meta-analysis included 27,729 PWH from 12 studies reporting the prevalence of fungemia in PWH after 1996. To our knowledge, this is the first systematic review and meta-analysis describing the global prevalence of fungemia in PWH after 1996. We found that the overall pooled prevalence of fungemia in PWH was 3.3%. There was, however, substantial heterogeneity between the included studies. Furthermore, this review revealed statistically significant differences in the prevalence of fungemia across different geographic regions and fungal pathogens. We found no significant difference in the prevalence of fungemia across different ART time periods (July 1996–September 2015 and September 2015–January 2025).

Our findings revealed statistically significant differences in the prevalence of fungemia in PWH across geographic regions, with the highest prevalence of 6.8% in Asia, 2.4% in South America, and lowest in Africa at 0.2%. There were no studies that reported the prevalence in European settings. The low prevalence estimate for Africa is surprising, as the Joint United Programme in HIV/AIDS (UNAIDS) estimated that in 2024, only 84% of all PWH in Eastern and Southern Africa were receiving ART, and that there were 260,000 AIDS-related deaths [33]. Thus, the low reported prevalence of fungemia among PWH in Africa reported in our review is likely an underestimation of the true burden in line with the low certainty of evidence, as well as a reflection of diagnostic limitations in this region. The diagnosis of fungemia in our study was limited to detection of fungal pathogens in blood cultures, which excluded other low-cost diagnostic methods such as antigen and serological detection, which may be the only diagnostic tests used in low-resource settings [34]. Additionally, environmental factors, such as higher temperatures, humid weather, and transmission pathways, also influence geographic differences, making PWH living in some regions more vulnerable to invasive fungal infections [35].

The most common fungal pathogen causing fungemia in PWH with a prevalence of 4.8% in our review was *Talaromyces marneffei*, a pathogen known to cause disseminated infections [36]. Prior studies have described the serious health threat that *Talaromyces marneffei* poses to PWH in Southeast Asia [37,38], and in our review, this pathogen was only described in studies that included data from this region. Along with our findings of the highest pooled prevalence of fungemia in Asia, these results underline substantial challenges with invasive fungal infections caused by *Talaromyces marneffei* in this region. The second most common pathogen causing fungemia in PWH in this review was *Histoplasma* spp. with a prevalence of 2.2%. Less frequently observed were *Cryptococcus* spp. and *Candida* spp. with prevalences of 0.9% and 0.6%, respectively. *Cryptococcus* spp. mainly causes meningitis, and cryptococcemia is often underdiagnosed due to the clinical symptoms involving the central nervous system [39]. However, a review presented that 47–70% of PWH with proven cryptococcal meningitis also had positive blood cultures, indicating that the disease is often disseminated with concurrent cryptococcemia [40]. This suggests that cryptococcemia may be underdiagnosed, explaining its low prevalence in our review.

Our subgroup analysis on different ART time periods (July 1996–September 2015 and September 2015–January 2025) revealed no significant difference in prevalence of fungemia in PWH after the change in treatment guidelines in September 2015. The guidelines recommending that all PWH initiate early treatment have improved viral suppression and contributed to better clinical outcomes for PWH [5,9]. Our findings are inconsistent with the increase in PWH receiving ART, and thus the decrease in PWH with advanced HIV disease [33]. This may be due to low quality of the included studies, publication bias, and small sample sizes, resulting in lack of full representativeness of PWH in this systematic review and meta-analysis. Despite the global widespread use of ART, gaps in HIV treatment remain. Four of the included studies only included PWH with low CD4+ T-cell count (<200 cells/μL or <100 cells/μL) [22,25,30,31], which represent PWH without viral suppression. We initially planned to determine the CD4+ T-cell count in PWH with fungemia as a secondary objective, but only three studies reported the median CD4+ T-cell count in those with fungemia [22,26,27]. The median CD4+ T-cell counts of the PWH with fungemia in the three studies were 14 cells/μL (range: 1–222), 3.5 cells/μL (range: 1–28), and 25 cells/μL (range: 4–166). This emphasizes that PWH with fungemia have severe immunosuppression. However, the data represent a small sample size, which limits the generalizability.

### Strengths and Limitations

This systematic review has some limitations. First, we excluded all non-English-language articles. Second, significant heterogeneity was present across the included studies, reducing the precision of our pooled estimates. The substantial heterogeneity also reduces the generalizability of the results. Third, this review only included culture-proven cases of fungemia, which might lead to an underestimation of the true global prevalence of fungemia, especially in low-resource settings. Fourth, substantial publication bias was revealed. Visual inspection of the funnel plot revealed asymmetry, with an overrepresentation of positive effects estimates, which may have resulted in imprecise and potentially overestimated pooled estimates. Likewise, the Egger’s test quantified significant publication bias. This pattern suggests that studies with no or low findings are underrepresented, limiting the representativeness of the pooled estimates. Last, the certainty of the evidence in all analyses was rated very low and the results should therefore be interpreted with caution.

Nevertheless, this comprehensive systematic review and meta-analysis is the first to provide an overview of the global burden of fungemia in PWH. This review was conducted using a comprehensive search string to identify as much relevant data as possible to conduct an overview of the current research on fungemia in PWH. Moreover, this systematic review and meta-analysis was conducted according to PRISMA guidelines to ensure the quality of this review. Also, a protocol was submitted before the review was conducted to enhance transparency and reduce bias.

## 5. Conclusions

In this systematic review and meta-analysis, our findings suggest that fungemia may be prevalent among PWH despite wide availability of ART. However, the pooled prevalence estimate of 3.3% was derived from studies with substantial heterogeneity and may therefore differ from the true global burden. Based on a limited number of studies, the available evidence is insufficient to determine whether the prevalence of fungemia changed following the change in ART treatment guidelines in September 2015. This finding was limited by the available evidence and should not be interpreted as evidence of absence of temporal changes.

The overall prevalence of fungemia among PWH varied statistically significantly across different geographic regions (Asia, Africa, and South America), and the burden of fungemia was highest in Asia, although this finding may be indicative of differential access to diagnostic tests. Likewise, the pathogen distribution varied, and the highest prevalence of fungemia in PWH was caused by *Talaromyces marneffei*, although this pathogen was limited to studies from Asia. However, the certainty of evidence of all results was rated very low, and the findings should be interpreted with caution. Our findings suggest that future research should support optimizing treatment for PWH to reduce the burden of invasive fungal infections.

## Figures and Tables

**Table 1:** Study characteristics of included studies.

Author and Year	Study Design	Study Period	Country	Sample Size ^a^ (n)	Cases of Fungemia (n)	Prevalence (%)	Prevalence Description
Falci D.R. (2019) [21]	Cohort	2016–2018	Brazil	570	11	1.9	Prevalence of fungemia caused by *Histoplasma* spp. in in-patient PWH with fever and one other symptom ^b^
Ferreira M.D.F (2019) [22]	Cohort	2015–2015	Brazil	108	4	3.7	Prevalence of cryptococcemia in in-patient PWH with CD4+ T-cell count ≤ 200 cells/mm^3^ or WHO stage III/IV
Gutierrez M.E. (2005) [23]	Cohort	1997–2003	Panama	2379	123	5.2	Prevalence of fungemia caused by *Histoplasma* spp. in in-patient PWH
Hoffmann E.R. (2016) [24]	Cohort	2014–2015	Brazil	78	1	1.3	Prevalence of fungemia caused by *Histoplasma* spp. in in-patient PWH with suspected disseminated histoplasmosis
Makadzange T.A. (2021) [25]	Cross-sectional	2015–2016	Zimbabwe	1597	10	0.6	Prevalence of cryptococcemia in PWH with CD4+ T-cell count ≤ 100 cells/mm^3^
Nguyen D. (2020) [26]	Cohort	2012–2016	French Guiana	369	10	2.7	Prevalence of fungemia caused by *Histoplasma* spp. in in-patient PWH
Qi T. (2016) [27]	Cohort	2009–2015	China	2442	97	4.0	Prevalence of fungemia in in-patient PWH
Rosas R.C (2003) [28]	Cohort	1997–1999	Brazil	111	9	8.1	Prevalence of fungemia in in-patient PWH with febrile illness (temp ≥ 38°) and late-stage AIDS
Shen Y.Z (2007) [29]	Cohort	2004–2006	China	85	4	4.7	Prevalence of fungemia in in-patient PWH
Van Schalkwyk E. (2020) [30]	Cross-sectional	2015–2016	South Africa	5266	1	0.02	Prevalence of fungemia in PWH with CD4+ T-cell count ≤ 100 cells/mm^3^
Wake R.M. (2019) [31]	Cohort	2015–2017	South Africa	7149	11	0.2	Prevalence of cryptococcemia in PWH with CD4+ T-cell count ≤ 100 cells/mm^3^ and tested for CrAg
Ying R.S. (2020) [32]	Cross-sectional	2011–2017	China	7575	696	9.2	Prevalence of fungemia caused by *Talaromyces marneffei* in in-patient PWH

^a^ Sample size of people with HIV (PWH); ^b^ Weight loss, diarrhea, and miliary pattern on thorax imaging, pancytopenia, splenomegaly, or hepatomegaly.

**Table 2:** Newcastle–Ottawa assessment scale, cross-sectional studies.

Reference	Q1	Q2	Q3	Q4 ^☆☆^	Q5 ^☆☆^	Q6 ^☆☆^	Q7	Overall Score	Score
Falci D.R. (2019) [21]	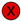	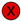	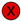	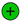 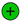	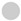	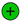 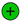	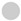	4/10	Low
Ferreira M.D.F. (2019) [22]	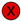	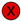	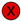	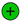 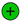	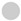	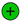 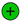	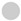	4/10	Low
Gutierrez M.E. (2005) [23]	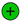	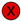	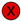	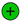 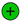	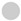	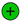 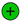	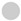	5/10	Moderate
Hoffmann E.R. (2016) [24]	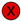	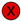	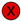	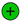 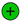	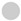	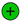 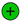	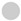	4/10	Low
Makadzange T.A. (2021) [25]	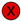	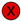	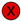	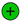 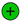	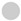	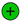 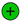	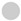	4/10	Low
Nguyen D. (2020) [26]	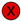	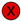	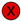	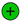 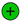	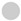	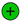 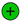	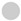	4/10	Low
Qi T. (2016) [27]	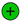	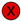	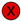	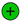 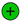	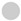	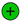 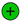	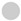	5/10	Moderate
Rosas R.C. (2003) [28]	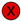	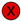	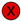	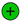 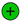	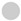	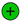 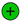	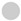	4/10	Low
Shen Y.Z (2007) [29]	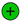	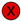	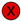	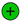 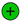	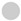	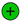 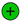	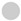	5/10	Moderate
Van Schalkwyk E. (2020) [30]	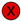	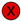	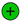	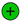 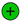	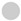	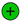 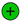	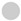	5/10	Moderate
Wake R.M. (2019) [31]	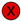	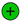	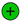	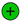 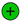	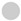	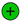 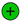	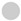	6/10	High
Ying R.S. (2020) [32]	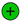	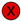	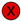	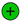 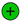	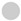	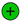 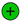	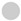	5/10	Moderate
		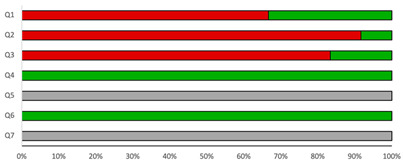							
								Judgement	
								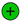	-
								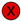	^☆^
								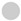	Not applicable


Table 2 Quality assessment of included studies conducted using Newcastle–Ottawa quality assessment scale for cross-sectional studies. Q1: Representativeness of the cases. Q2: Sample size. Q3: Non-response rate. Q4: Ascertainment of the exposure. Q5: The potential confounders were investigated by subgroup analysis or multivariable analysis. Q6: Assessment of the outcome. Q7: Statistical test. ^☆☆^ 2 points achievable.

## Data Availability

No new data were created or analyzed in this study. Data sharing is not applicable to this article.

## Supplementary Materials

The following supporting information can be downloaded at https://www.mdpi.com/article/10.3390/microorganisms14010225/s1. Table S1: Full search strategy; Table S2: Joanna Briggs institute checklist for prevalence studies [21,22,23,24,25,26,27,28,29,30,31,32]; Figure S1: Baujot plot; Figure S2: Influential study diagnostics [21,22,23,24,25,26,27,28,29,30,31,32]; Figure S3: Funnel plot.

## Abbreviations

The following abbreviations are used in this manuscript:

HIV	Human immunodeficiency virus
PWH	People with HIV
ART	Antiretroviral therapy
WHO	World Health Organization
JBI	Joanna Briggs Institute
PRISMA	Preferred Reporting Items for Systematic Reviews and Meta-Analysis
PROSPERO	Prospective Register for Systematic Reviews
NOS	Newcastle–Ottawa scale
GRADE	Grades of recommendation, assessment, development, and evaluation
CI	Confidence interval
UNAIDS	Joint United Programme in HIV/AIDS
