# Supplementary material for: Prevalence of Fungemia in People with HIV: A Systematic Review and Meta-Analysis
Source: Microorganisms. 2026 Jan 19;14(1):225. doi: 10.3390/microorganisms14010225 (PMC12844278; doi:10.3390/microorganisms14010225)
Supplement: Supplementary file 1 [file microorganisms-14-00225-s001.zip › microorganisms-4042329-supplementary.pdf]

Supplementary Materials:

Table S1 Full search strategy

DATABASE	SEARCHES	RESTRICTIONS
**PUBMED**	("HIV seropositiv*" OR "HIV/AIDS" OR "hiv infection*" OR "human immunodeficiency virus" OR HIV OR PWH OR PWHIV OR PWH OR AIDS OR "Acquired immuno deficiency syndrome" OR "Acquired immune-deficiency syndrome") AND ("fungal septi*" OR "fungal bloodstream infection*" OR fungemia OR fungaemia OR "invasive fungal infection*" OR "invasive myco*" OR "systemic myco*" OR "Disseminated fungal infection*" OR "invasive mold infection*" OR Candidemia OR (Histoplasmosis OR Cryptococcus OR Coccidiomycosis OR Aspergillus AND blood)) OR (("HIV"[Mesh] OR "HIV Infections"[Mesh]) AND ("Invasive Fungal Infections"[Mesh]))	English language. Publication dates: 1996-2025
**EMBASE**	1. exp systemic mycosis/<br>2. exp human immunodeficiency virus/<br>3. (("HIV seropositiv*" or "HIV/AIDS" or "hiv infection*" or "human immunodeficiency virus" or HIV or PWH or PWHIV or PWH or AIDS or "Acquired immuno deficiency syndrome" or "Acquired immune-deficiency syndrome") and ("fungal septi*" or "fungal bloodstream infection*" or fungemia or fungaemia or "invasive fungal infection*" or "invasive myco*" or "systemic myco*" or "Disseminated fungal infection*" or "invasive mold infection*" or Candidemia or ((Histoplasmosis or Cryptococcus or Coccidiomycosis or Aspergillus) and blood))).ab,kf,ti.[mp=title, abstract, heading word, drug trade name, original title, device manufacturer, drug manufacturer, device trade name, keyword heading word, floating subheading word, candidate term word]<br>4. 1 and 2<br>5. 3 or 4	English language. Publication dates: 1996-2025 Restricted to search in abstract, title and keywords
**WEB OF SCIENCE**	("HIV seropositiv*" OR "HIV/AIDS" OR "hiv infection*" OR "human immunodeficiency virus" OR HIV OR PWH OR PWHIV OR PWH OR AIDS OR "Acquired immuno deficiency syndrome" OR "Acquired immune-deficiency syndrome") AND ("fungal septi*" OR "fungal bloodstream infection*" OR fungemia OR fungaemia OR "invasive fungal infection*" OR "invasive myco*" OR "systemic myco*" OR "Disseminated fungal infection*" OR "invasive mold infection*" OR Candidemia OR (Histoplasmosis OR Cryptococcus OR Coccidiomycosis OR Aspergillus AND blood))	English language. Publication dates: 1996-2025 Restricted to search in abstract
**CENTRAL - COCHRANE CENTRAL REGISTER OF**	("HIV seropositiv*" OR "HIV/AIDS" OR "hiv infection*" OR "human immunodeficiency virus" OR HIV OR PWH OR PWHIV OR PWH OR AIDS OR "Acquired immuno deficiency syndrome" OR "Acquired immune-deficiency syndrome") AND ("fungal septi*" OR "fungal bloodstream	English language. Publication dates: 1996-2025 Restricted to search in title, abstract and keywords

**CONTROLLED TRIALS**	infection*" OR fungemia OR fungaemia OR "invasive fungal infection*" OR "invasive myco*" OR "systemic myco*" OR "Disseminated fungal infection*" OR "invasive mold infection*" OR Candidemia OR (Histoplasmosis OR Cryptococcus OR Coccidiomycosis OR Aspergillus AND blood))	
**SCOPUS**	("HIV seropositiv*" OR "HIV/AIDS" OR "hiv infection*" OR "human immunodeficiency virus" OR HIV OR PWH OR PWHIV OR PWH OR AIDS OR "Acquired immuno deficiency syndrome" OR "Acquired immune-deficiency syndrome") AND ("fungal septi*" OR "fungal bloodstream infection*" OR fungemia OR fungaemia OR "invasive fungal infection*" OR "invasive myco*" OR "systemic myco*" OR "Disseminated fungal infection*" OR "invasive mold infection*" OR Candidemia OR (Histoplasmosis OR Cryptococcus OR Coccidiomycosis OR Aspergillus AND blood))	English language. Publication dates: 1996-2025 Restricted to search in title, abstract and keywords

Table S2 Joanna Briggs institute checklist for prevalence studies

Reference	Q1	Q2	Q3	Q4	Q5	Q6	Q7	Q8	Q9
Falci D.R. (2019)	X	+	+	+		+	+		-
Ferreira M.D.F. (2019)	X	+	X	+		+	+		-
Gutierrez M.E. (2005)	+	+	+	+		+	-		X
Hoffmann E.R. (2016)	X	+	X	+		+	+		-
Makadzange T.A. (2021)	X	+	+	+		+	-		X
Nguyen D. (2020)	X	+	+	+		+	-		-
Qi T. (2016)	+	+	+	+		+	-		-
Rosas R.C. (2003)	X	+	X	+		+	-		-
Shen Y.Z (2007)	+	+	X	+		+	-		-
Van Schalkwyk E. (2020)	X	+	+	+		+	+		+
Wake R.M. (2019)	X	+	+	+		+	+		+
Ying R.S. (2020)	+	+	+	+		+	-		-

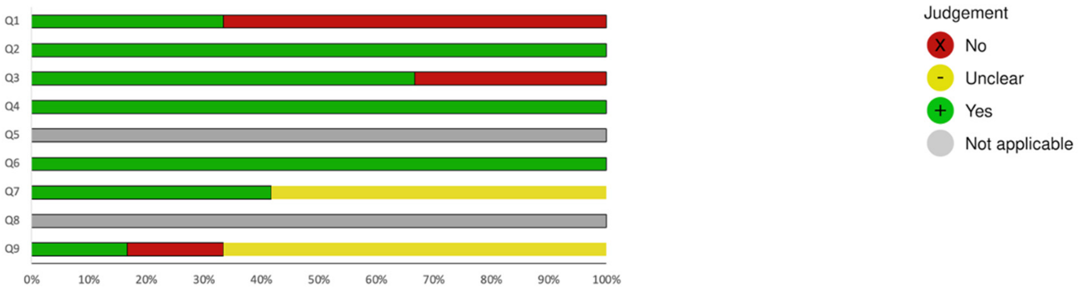

Table S2 Quality Assessment of included studies conducted using Joanna Briggs institute checklist for prevalence studies. Q1: Was the sample frame appropriate to address the target population? Q2: Were study participants recruited in an appropriate way? Q3: Was the sample size adequate? Q4: Were the study subjects and setting described in detail? Q5: Was data analysis conducted with

sufficient coverage of the identified sample? Q6: Were valid methods used for the identification of the condition? Q7: Was the condition measured in a standard, reliable way for all participants? Q8: Was there appropriate statistical analysis? Q9: Was the response rate adequate, and if not, was the low response rate managed appropriately?

Figure S1 Baujot plot

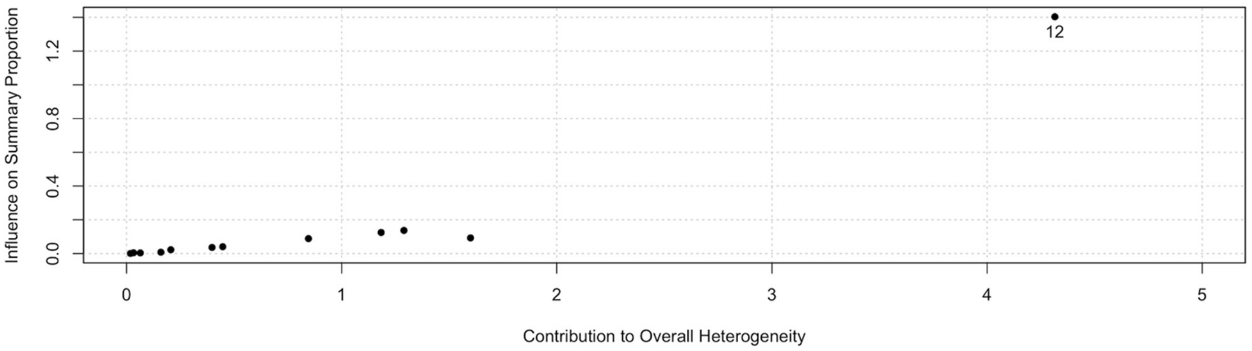

Figure S1 Baujot plot showing the influence on Summary Proportion and Contribution to Overall Heterogeneity. Study 12: Ying R.S. et al. 2020.

Figure S2 Influential study diagnostics

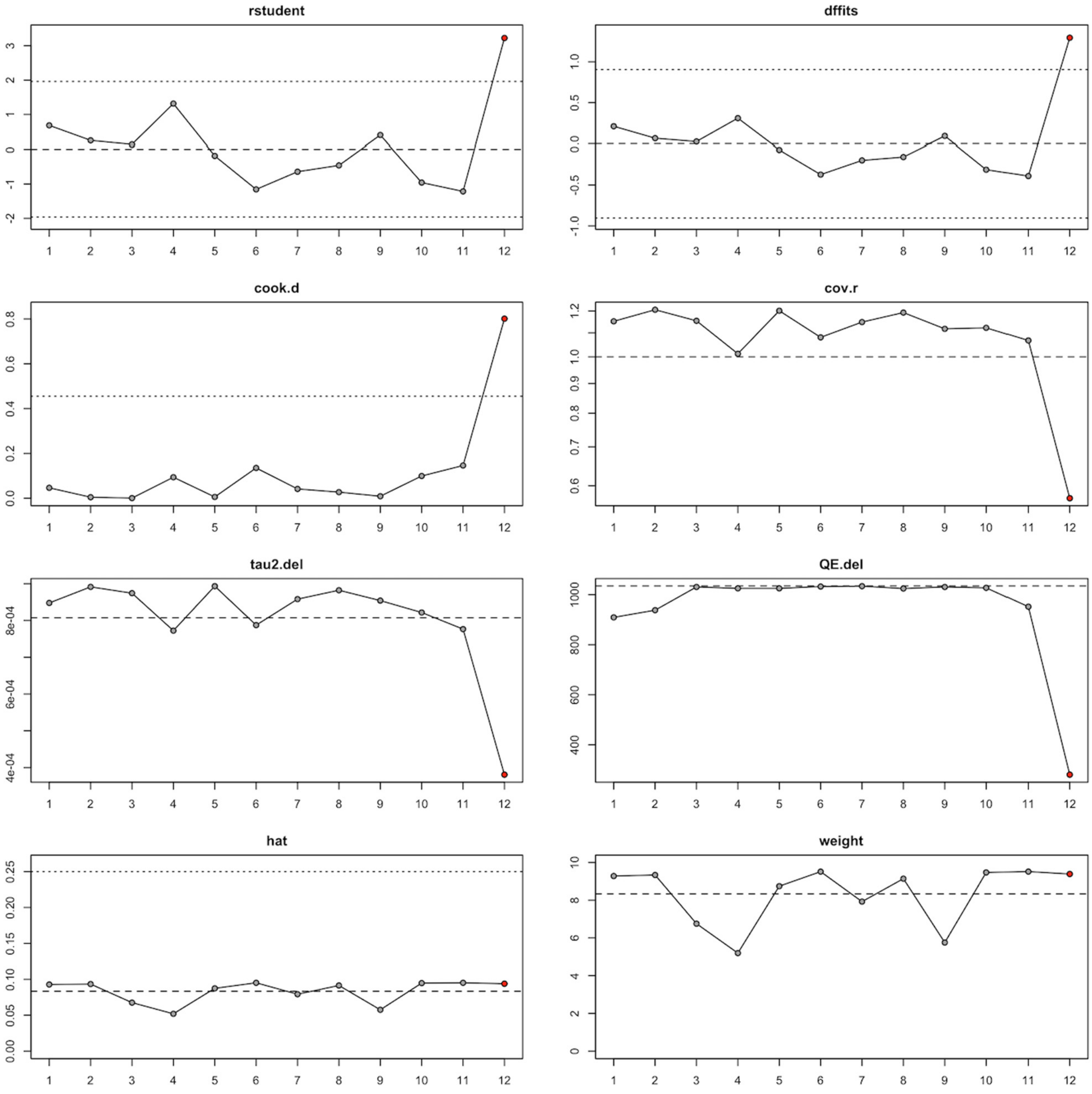

Figure S2 Influent study diagnostics of included studies. Rstudent: externally standardized residuals; dffits: DFFITS values; cook.d: cook's distance; cov.r: covariance ratios; tau2.del: leave-one-out estimates of the amount of heterogeneity, QE.del: leave-one-out values of the covariance ratios; hat: hat values; weight: weight.

Figure S3 Funnel plot

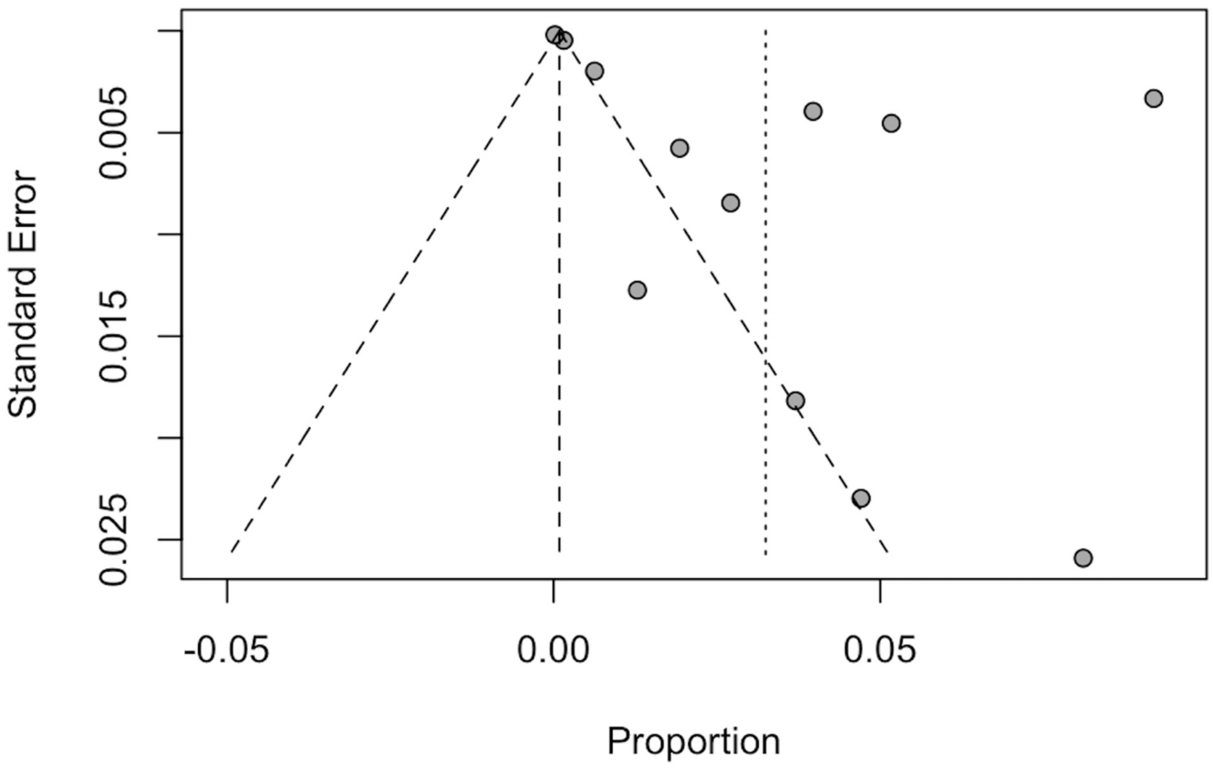

Figure S3 Funnel plot of included studies.
